# Evaluation of community pharmacies regarding dispensing practices of antibiotics in two districts of central Nepal

**DOI:** 10.1371/journal.pone.0183907

**Published:** 2017-09-26

**Authors:** Mukhtar Ansari

**Affiliations:** Department of Clinical Pharmacy, College of Pharmacy, University of Hail, Hail, Saudi Arabia; Natural Environment Research Council, UNITED KINGDOM

## Abstract

**Objective:**

To evaluate the status of community pharmacies, their staff, and practices toward dispensing antibiotics.

**Design:**

Cross-sectional, prospective.

**Place and duration of study:**

Community pharmacies in two districts of central Nepal, from March 2016 to May 2016.

**Methods:**

A systematic random sampling approach was adopted to sample 161 community pharmacies. Data on the registration status of pharmacies, qualification or training of dispensing staff, and the practice of dispensing antibiotics were collected using a pre-tested questionnaire. Face to face interviews were carried out by a previously trained interviewer. Data were analyzed for descriptive and inferential statistics using IBM SPSS Statistics 21.

**Results:**

Among 161 community pharmacies, 25% were not registered and most of them were located in rural areas. It was typical (66.5%) to dispense antibiotics without prescription and most (91.4%) of the staffs involved in dispensing were non-pharmacists. Furthermore, the study revealed common practices of replacing one brand of antibiotic with other brands (66%), dispensing incomplete courses of antibiotics (73%), and not giving any advice regarding antibiotic use (39%) or completion of a full course of therapy (80%). There were significant (p < 0.001) relationships between the location of pharmacies (rural vs urban) and the qualifications of the pharmacy staff.

**Conclusion:**

Dispensing antibiotics without prescription and by non-pharmacists are common in this region. The study also found several issues regarding the irrational use of antibiotics. Thus, there is an urgent need to address these issues and promote the informed use of antibiotics.

## Introduction

A community pharmacy is a healthcare facility responsible for providing pharmaceutical care services to the community[1]. A “Community Pharmacy” is a professional term and often referred to as a medical shop or store (*Aushadhi pashal*) or a retail pharmacy in Nepal.

In Nepal, the professional role of pharmacists is not well established [2]. Moreover, people consider community pharmacists as chemists or medicine traders. Consumers’ behavior in purchasing medicine from medical stores is similar to buying food items or general commodities from a grocery store. In developing countries like Nepal, the majority of people reside in rural areas where healthcare facilities are scarce [3]. Thus, community pharmacies have become the most favored place for those seeking healthcare for general ailments [4, 5]. Furthermore, consultation is easier and cheaper [6]. However, the problem is non-professionals, i.e., non-pharmacists, who operate community pharmacies, especially in rural areas. Indeed, the delivery of healthcare by non-professionals can be detrimental in certain instances [2].

Antibiotics are highly prescribed and frequently used to treat infections. Proper disease diagnoses and the rational selection of antibiotics are crucial. Certain infectious diseases are self-limiting, such as viral rhinitis, and thus there is no use for an antibiotic treatment. Similarly, antibiotics are less effective for the treatment of diarrhea caused by viral infections. The major complication due to uncontrolled use of antibiotics is the development of antibiotic-resistant bacterial strains that further complicate treatment [7]. In developing countries, there is a lack of strict guidelines or policies on the rational use of antibiotics, which often results in the overuse or unnecessary use of antibiotics. The Government of Nepal has formulated the National Antibiotic Treatment Guidelines 2014 [8]. However, the implementation of these guidelines remains a challenge. Thus, one can easily envisage the level of irrational antibiotic use in Nepal.

The present study aimed to evaluate the status of community pharmacies, their staff, and their practices of dispensing antibiotics. The Institution Review Board of National Medical College, Birgunj, Nepal approved this study.

## Materials and methods

### Design and setting

This was a cross-sectional prospective study carried out in the Bara and Parsa districts of central Nepal from March 2016 to May 2016.

### Study population

The study population included 276 community pharmacies in the above two districts listed in the updated directory from the seventh session (2013) (fiscal year 2012/2013) of the Nepal Chemists and Druggists Association (NCDA), Narayani, Nepal.

### Sample size and sampling procedure

The Raosoft sample size calculator was used to calculate a required sample size of 161 based on a population size of 276 with a 5% margin of error, 95% confidence level, and 50% response distribution [9].

A systematic random sampling approach was adopted to sample the pharmacies. The study population was determined using the NCDA directory. In each of the two districts, the first listed pharmacy was set as the starting point for interviews of the dispensing staff. Maintaining an alternate number approach, pharmacies reluctant to participate in the study were exempted from the study and the next pharmacy was contacted.

### Instruments and instrumentation

A self-designed questionnaire was prepared based on the study objectives. The questionnaire was pre-tested through piloting among 10 randomly selected pharmacies, including those located in rural areas. Feedback from the pilot questionnaire was used to modify the options for educational background and dispensing practices regarding antibiotics. Next, the modified questionnaire was distributed to the sample population (S1 File). A previously trained bilingual (*Nepali and Bhojpuri*) interviewer was employed to carry out interviews during peak (day) time, i.e. from 9 am to 4 pm, and the study was conducted under the close supervision of the researcher. Nepali and Bhojpuri are the two main languages used for communication in this part of Nepal. Information about prescriptions was obtained indirectly through interviewing the pharmacy personnel. Before conducting the interviews, a written informed consent was obtained from each participant. Only the members of staff who agreed to participate in the study were interviewed. During pilot study, only one dispensing staff from each pharmacies participated. Furthermore, majority of the pharmacies had single dispensing staff. Thus, only one dispensing staff from each pharmacy was interviewed.

### Outcomes, measures, and data analyses

Data were analyzed for descriptive and inferential statistics using IBM SPSS Statistics 21. The results were expressed as counts and percentages. Associations were tested using the Chi-square test.

## Results

In 83% of the community pharmacies, only one dispensing member of staff was present at the time of the interview. Among the 161 community pharmacies surveyed, 45% were from the Bara district and 55% were from the Parsa district of Nepal. Bara and Parsa are two districts in the central plain (Terai) region of Nepal, which adjoins the northern Indian border of the Bihar province. People of these regions can easily visit both countries (i.e., Nepal and India) for healthcare and other social needs without any restriction.

More than two-thirds (68%) of the community pharmacies were urban based. Nearly two-thirds (63.6%) of the pharmacy staff with a Community Medical Assistant (CMA) degree worked in rural areas. There was a significant relationship between the pharmacy location (rural vs. urban) and the qualification (Table 1) of pharmacy staff (p < 0.001). Dispensing practices such as dispensing antibiotics without prescription was significantly associated with age of the dispensing staffs (p < 0.043) and years of dispensing experience (p < 0.001). However, gender didn’t make significant difference in dispensing antibiotics irrationally. Similarly, taking feedback after dispensing antibiotics and years of dispensing experience was significantly associated (p = 0.003).

**Table 1:** Demographic characteristics of pharmacy staff (n = 161).

Characteristics		Number	Percentage
Gender	Male	144	89.4
	Female	17	10.6
Age	Upto 30 yrs	74	46
	31–40 yrs	46	28.6
	41–50 yrs	28	17.4
	> 50 yrs	13	8.1
Work experience	< 5 yrs	33	20.5
	5–10 yrs	49	30.4
	> 10yrs	79	49.1
Qualification	Primary	52	32.3
	Secondary	60	37.3
	Higher secondary	13	8.1
	D Pharm	9	5.5
	B Pharm	5	3.1
	CMA	22	13.7

Almost two-thirds (62.1%) of the community pharmacies were registered with the Department of Drug Administration (DDA), and the basis of their registration was orientation training. Among these pharmacies, 79% were in urban areas of the two districts. On the other hand, 24.9% of the pharmacies were not registered at all, and 77% of this subgroup was located in the countryside. There was a significant relationship between the location of the pharmacy (rural vs. urban) and its registration status (registered vs. not registered) (p < 0.001). Similarly, all “pharmacists borrowed licenses” and “pharmacists owned licenses” were in cities.

The term “pharmacist’s borrowed license” used in this study indicates that the registered pharmacist’s educational qualification and Nepal Pharmacy Council’s registration certificate were supplied to the DDA at the time of registration of the pharmacy. This practice is a result of an agreement between the pharmacy owner and the pharmacist; however in reality, the pharmacist does not work in the pharmacy but rather works somewhere else. This agreement fulfills legal formalities, and in return, the owner pays the pharmacist. In contrast, a “pharmacists owned license” implies that the pharmacy is registered with the DDA under the name of the pharmacist; i.e., the pharmacist owns and operates the pharmacy. The registration status of community pharmacies is depicted in Table 2.

**Table 2:** Registration status of community pharmacies (n = 161).

Registration status	Number	Percentage
Orientation training	100	62.1
Pharmacist's borrowed licence	16	9.9
Pharmacist's own registered pharmacy	5	3.1
Not registered	40	24.9

Approximately 70% of prescriptions were for a single antibiotic, whereas the remaining 30% consisted of two antibiotics (Table 3). Dispensing antibiotics without prescription was common (66.5%). Furthermore, in 76% of such cases, the dispensing staff strongly persuaded their customers to buy antibiotics. In only 24% of these cases did the customers ask for antibiotics of their own accord. After dispensing antibiotics, 56% of the pharmacy staff reported that they did not consider it worth it to ensure patients understood the instructions regarding the proper use of antibiotics.

**Table 3:** Number of antibiotics dispensed daily.

Quantity of antibiotics dispensed	Number of pharmacy	Percentage of pharmacy
1 to 10	24	14.9
11 to 20	67	41.6
21 to 30	16	9.9
31 to 40	9	5.6
41 to 50	12	7.5
51 to 250	33	20.5

Two-thirds (66%) of the staff stated that they replaced one brand of antibiotic with another if the prescribed brand was not available. Similarly, 15% and 19% of the staff mentioned that they recommended patients to visit other pharmacies and the prescriber respectively if the prescribed brand was not available in their pharmacy. Dispensing a reduced quantity (i.e., number of tablets, capsules, etc.) of antibiotics was frequently observed (73%). The study also revealed that pharmacists often replaced the prescribed brand with a lower-priced one (27%) but the brand substituted belonged to the same medicine.

Thirty-eight percent of the pharmacy staff gave advice to their patients about the importance of completing the full course of antibiotics. Only three percent emphasized to adhere to the dosage regimen of the antibiotic therapy. Twenty percent of the staff advised patients to follow both the completion of the full course of therapy and adhere to the dosage regimen. Interestingly, 39% of the dispensing staff did not give any advice to their patients regarding the rational use of antibiotics.

**Fig 1:**
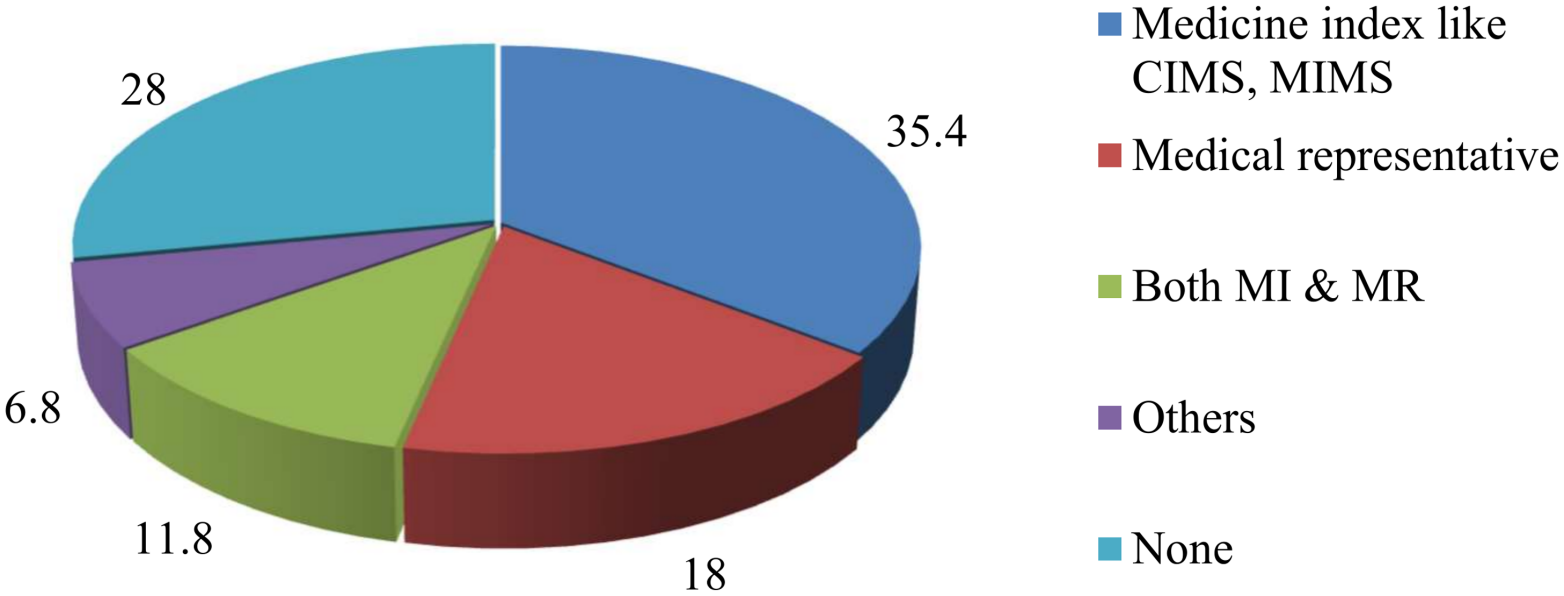
Sources of medicine information for pharmacy staffs.

Twenty-eight percent of the dispensing staff had not studied any information sources regarding dispensed medicines to improve their current knowledge. Among the staff who researched medicine information, 35.4% opted for medical indexes, such as the Current Index of Medical Specialties (CIMS) and Monthly Index of Medical Specialties (MIMS). Likewise, 18% of this subgroup trusted medical representatives as a relevant source of medicine information “Fig 1”.

**Fig 2:**
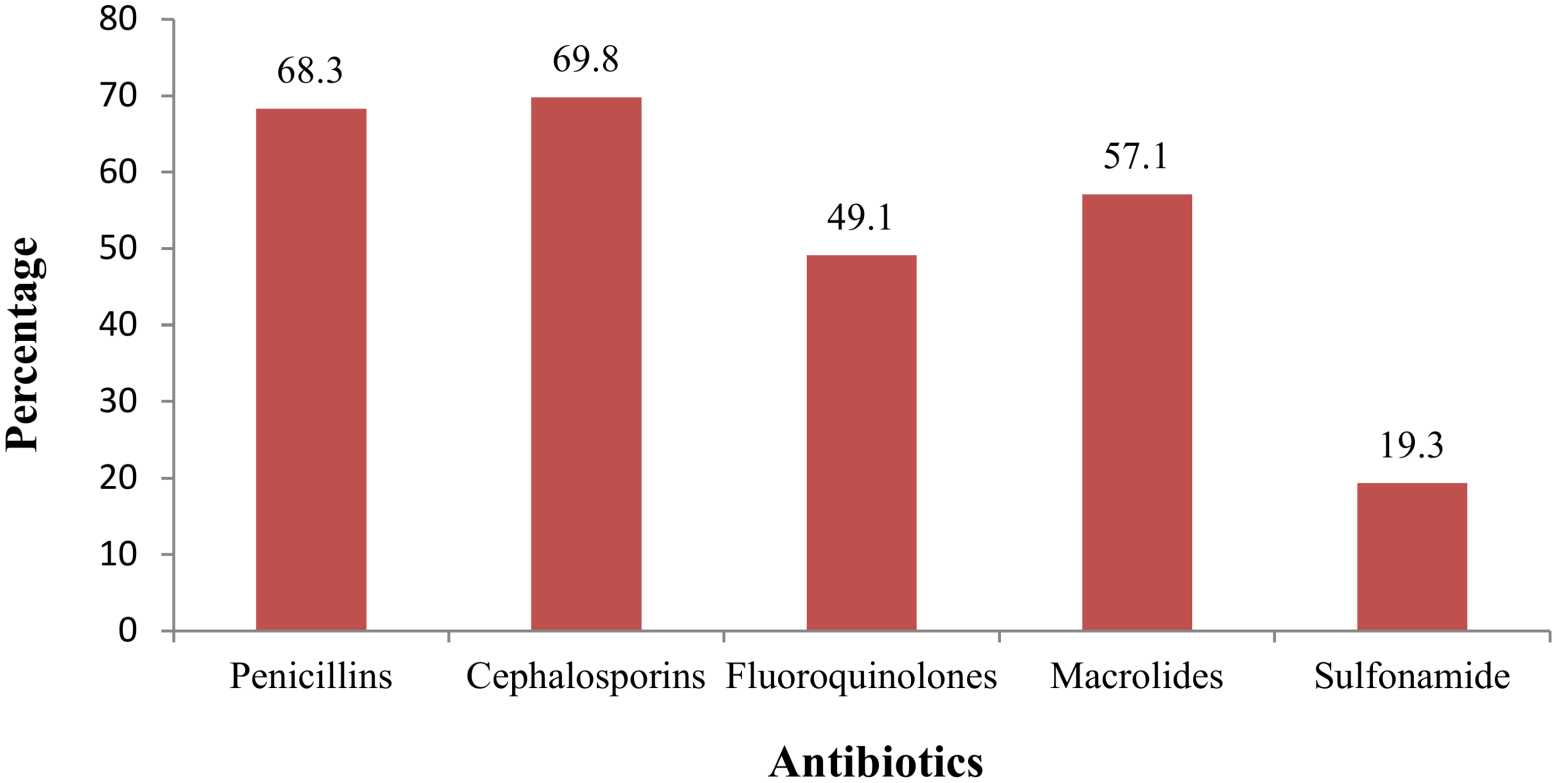
Dispensing pattern of antibiotics.

The dispensing pattern of antibiotics revealed that cephalosporins were the most frequently dispensed antibiotics, followed by penicillins, macrolides, fluoroquinolones and sulfonamide “Fig 2”. The antibiotics prescribed and dispensed, dispensed without prescription, or demanded by customers were mainly intended for the treatment of respiratory tract complications (e.g. cough), fever, and urinary tract infections.

## Discussion

The present study demonstrated various components related to the irrational dispensing of antibiotics in the community. In this study, the terms “pharmacy staff” and “dispensing staff,” rather than pharmacists have preferably been used because the staff involved in dispensing antibiotics were mostly non-professionals (non-pharmacists) [2, 10].

Although the level of educational qualification is low among the pharmacy staff in rural settings, healthcare professionals, such as CMAs, prefer to work in this location. There is a short supply of graduate physicians; therefore, CMAs often perform dual roles (i.e., prescriber as well as dispenser) [11]. CMAs are basic level health cadres responsible for providing primary healthcare to the public, mainly in rural communities, through primary healthcare centers [12]. CMA is a three years diploma or certificate level health education program in Nepal. People after passing class 10 during basic schooling or school leaving certificate (SLC) become eligible to enroll in this course.

Among the pharmacies surveyed, the presence of registered pharmacists was very limited (<9%). Pharmacists are the trained medicine expert and they can contribute significantly toward promoting rational use of medicine in the community[13]. Additionally, despite the availability of the National Antibiotic Treatment Guideline in 2014[14], its recommendations have not been implemented. Not surprisingly, a lack of enforced policies can lead to the irrational use of antibiotics. The DDA of the Ministry of Health, Government of Nepal started a short (45 hours) training course in 1981 known as “orientation training” available to the public as a pre-requisite to register community pharmacies. The course was applicable to people with a basic education [15]. Subsequently, the duration of the course was increased to three months as per the need; however, this course no longer exists. During the lifetime of this training course, the number of pharmacists in the country was very limited[16], and the Government of Nepal had no choice than to offer the training opportunity. However, the people’s interest in pharmacy education and the number of pharmacy graduates subsequently grew, allowing the Government to discontinue the course. Therefore, the majority (79%) of long-standing community pharmacies were registered via orientation training. In contrast, 77% of the community pharmacies in villages were not registered with the DDA. This shows a lack of monitoring from the relevant authorities at the rural level.

Despite the legal requirement to have a registered pharmacist on duty at all times, in reality, the pharmacies are operated by unauthorized staff in many Asian countries [17, 18]. In our study, pharmacists were not found on duty at the time of dispensing but their names were present in legal documents, mainly due to the rule enforced by the Government of Nepal to have at least one registered pharmacist to operate and dispense new pharmacies [19]. In contrast, study conducted by Gokcekus et al found that majority (73.3%) of prescriptions were dispensed by pharmacists [20].

Approximately 70% of the prescriptions were for single antibiotics. This finding aligns with the study conducted in Yemen by Alshakka et al who reported 65% of single antibiotic prescriptions [21]. These results are an indication of good prescribing habits in terms of the number of antibiotics prescribed per prescription. In two-thirds of cases, however, the dispensing staffs were providing antibiotics without a prescription. A multi-center study conducted in Yemen and Uzbekistan by Belkina et al reported a higher percentage (78%) of antibiotic use without prescription [22]. An even higher percentage (88–91%) was reported in a study conducted by Nga et al in Vietnam [23]. Similarly, 56% of the pharmacy staff did not bother to ask for feedback from the patients. Our study found that 62% of patients did not seek advice from the pharmacy staff about the importance of completing the full course of antibiotic therapy. A systematic review by Miller and Goodman about the performance of community pharmacies in low and middle income Asian countries highlighted the above issues as major determinants of the irrational use of antibiotics [5]. As in other developing nations, Nepalese pharmacies resemble retail businesses operating within a competitive marketplace. When patients lack sufficient money to buy a complete course of antibiotics, they can often (73%) get a reduced quantity (i.e., number of tablets, capsules, etc.) of antibiotics. If this method did not work, the pharmacy staff preferred to replace the recommended brand with another cheaper brand. This finding shows the commercial attitude of community pharmacy staff. In reality, a community pharmacy should be less like a business and more like a professional service provider [24, 25].

A lack of updating their knowledge about antibiotic use was another main drawback among pharmacy staff. We revealed that 28% of the dispensing staff did not use any resource for medicine information. However, 34% of the staff used medical indexes, such as the CIMS and MIMS, and 18% used Medical Representatives (MRs) as sources of information about medicine use. Moreover, medical indexes found in pharmacies were outdated, and the MRs were mostly from a non-technical educational background. Although a two-and-a-half-day refresher course is advisable and conducted in collaboration with the NCDA and DDA on a regular basis to update the knowledge of dispensing staff, this training is not sufficient. Course participants are mostly pharmacy owners and not the actual dispensing staff; furthermore, the training was mainly urban based [26]. Thus, the refresher training is less beneficial because the opportunities intended for actual dispensing staff were often instead utilized by pharmacy owners.

The most common complications in which antibiotics were used included respiratory tract complications (e.g., cough), fever, and urinary tract infections. An Indian study by Ahmad et al also reported a similar finding [27]. Beta-lactam antibiotics, mainly cefixime and amoxicillin alone or in combination, were the most frequently dispensed antibiotics [28]. Ofloxacin and azithromycin were also among the frequently dispensed antibiotics.

Several reports show that irrational use of antibiotics is a worldwide problem. The problem is more pronounced in developing nations where there is lack of antibiotic use policy or the implementation part of such policy is poor. This study involved community pharmacies of two districts of Nepal only. Although these poor antibiotic dispensing practices are assumed to be consistent across the nation, multi-center studies are needed to confirm the generalizability of our findings.

## Conclusions

The status of the community pharmacies was miserable in terms of Good Pharmacy Practice. Although various factors can contribute to widespread irrational dispensing of antibiotics, lack of professionally trained personnel like pharmacists is crucial. Thus, there is an urgent need to address these issues and promote the rational use of antibiotics in the community. The Government of Nepal and the concerned authority should adopt strict strategies to monitor dispensing practices in the pharmacies and take necessary action.

## Supporting information

S1 File Survey questionnaire. (DOC)

## References

[1] About community pharmacy. Pharmaceutical Services Negotiating Committee. Accessed 13 Dec 2015. http://psnc.org.uk/psncs-work/about-community-pharmacy/ [Internet].

[2] Bhuvan KC, Alrasheedy AA, Ibrahim MIM. Do community pharmacists in Nepal have a role in adverse drug reaction reporting systems? Australas Med J. 2013;6(2):100–3. doi: 10.4066/AMJ.2013.1544 23483017 10.4066/AMJ.2013.1544 PMC3593519

[3] Nepal Demographic and Health Survey 2011. Kathmandu, Nepal: Ministry of Health and Population, New ERA, and ICF International, Calverton, Maryland http://dhsprogram.com/pubs/pdf/FR257/FR257%5B13April2012%5D.pdf [Internet]. 2012.

[4] Gyawali S, Rathore DS, Adhikari K, Shankar PR, KC VK, Basnet S. Pharmacy practice and injection use in community pharmacies in Pokhara city, Western Nepal. BMC Health Services Research. 2014;14(190). 10.1186/1472-6963-14-190 PMC4101856 24774195

[5] Miller R, Goodman C. Performance of retail pharmacies in low- and middle-income Asian settings: a systematic review. Health Policy and Planning. 2016 doi: 10.1093/heapol/czw007 26962123 10.1093/heapol/czw007 PMC4977427

[6] Hadi MA, Karami NA, Al-Muwalid AS, Al-Otabi A, Al-Subahi E, Bamomen A, et al Community pharmacists' knowledge, attitude, and practices towards dispensing antibiotics without prescription (DAwP): a cross-sectional survey in Makkah Province, Saudi Arabia. International Journal of Infectious Diseases. 2016;47:95–100. doi: 10.1016/j.ijid.2016.06.003 27343987 10.1016/j.ijid.2016.06.003

[7] Ansari M, Shah SVA, Sen A, Shekh S, Singh GK, Parajuli KP. A retrospective analysis of culture sensitivity and antimicrobial prescribing pattern in a teaching hospital of eastern Nepal. National Journal of Laboratory Medicine. 2012;1(1):29–33.

[8] National antibiotic treatment guidelines. Ministry of Health and Population, Government of Nepal, Kathmandu, Nepal. http://www.mohp.gov.np/images/pdf/guideline/National_Antibiotic_Treatment_Guidelines.pdf [Internet]. 2014.

[9] Sample size calculator. http://www.raosoft.com/samplesize.html [Internet]. [cited Dec 05, 2015].

[10] Poudel A, Khanal S, Alam K, Palaian S. Perception of Nepalese community pharmacists towards patient counseling and continuing pharmacy education program: A multicentric study. Journal of Clinical and Diagnostic Research. 2009;3(2):1408–13.

[11] Gupta RP, Ghimire J, Mahato RK, Kumal AB, BC RK, Bishwakarma DK, et al Human resource for health production capacity in Nepal: A glance. J Nepal Health Res Counc. 2013;11(24):144–8. 24362602

[12] Luitel NP, Jordans MJD, Adhikari A, Upadhaya N, Hanlon C, Lund C, et al Mental health care in Nepal: current situation and challenges for development of a district mental health care plan. Conflict and Health. 2015;9:3 doi: 10.1186/s13031-014-0030-5 25694792 10.1186/s13031-014-0030-5 PMC4331482

[13] Toklu HZ. Promoting evidence-based practice in pharmacies. Integrated Pharmacy Research and Practice. 2015;4:127–31. https://doi.org/10.2147/IPRP.S70406. 10.2147/IPRP.S70406 PMC5741015 29354526

[14] Palaian S, Ibrahim MIM, Mishra P. Pattern of adverse drug reactions reported by the community pharmacists in Nepal. Pharmacy Practice. 2010;8(3):201–7. 25126141 10.4321/s1886-36552010000300008 PMC4127056

[15] Kafle KK, Gartoulla RP, Pradhan YM, Karkee SB, Quick JD. Drug retailer training: experiences from Nepal. Soc Sci Med. 1992;35(8):1015–25. 1411696 10.1016/0277-9536(92)90241-h

[16] WHO. World health statitstics. http://www.who.int/whosis/whostat/EN_WHS2011_Full.pdf?ua=1 2011.

[17] Seeberg J. Connecting pills and people: an ethnography of the pharmaceutical nexus in Odisha, India. Med Anthropol Q. 2012;26(2):182–200. Epub 2012/08/22. . 22905436 10.1111/j.1548-1387.2012.01200.x

[18] Vu DH, van Rein N, Cobelens FG, Nguyen TT, Le VH, Brouwers JR. Suspected tuberculosis case detection and referral in private pharmacies in Viet Nam. Int J Tuberc Lung Dis. 2012;16(12):1625–9. Epub 2012/11/08. doi: 10.5588/ijtld.12.0295 . 23131260 10.5588/ijtld.12.0295

[19] Lowe RF, Montagu D. Legislation, regulation, and consolidation in the retail pharmacy sector in low income countries. Southern Med Review. 2009;2(2):35–44.

[20] Gokcekus L, Toklu HZ, Demirdamar R, Gumusel B. Dispensing practice in the community pharmacies in the Turkish Republic of Northern Cyprus. International journal of clinical pharmacy. 2012;34(2):312–24. Epub 2012/01/21. doi: 10.1007/s11096-011-9605-z . 22262499 10.1007/s11096-011-9605-z

[21] Alshakka M, Said K, Babakri M, Ansari M, Aldhubhani Adel., Hassali MA, et al A study on antibiotics prescribing pattern at outpatient department in four hospitals in Aden-Yemen. Journal of Pharmacy Practice and Community Medicine. 2016;2(3):88–93. http://dx.doi.org/10.5530/jppcm.2016.3.5.

[22] Belkina T, Al Warafi A, Hussein Eltom E, Tadjieva N, Kubena A, Vlcek J. Antibiotic use and knowledge in the community of Yemen, Saudi Arabia, and Uzbekistan. J Infect Dev Ctries. 2014;8(4):424–9. Epub 2014/04/15. doi: 10.3855/jidc.3866 . 24727507 10.3855/jidc.3866

[23] Nga do TT, Chuc NT, Hoa NP, Hoa NQ, Nguyen NT, Loan HT, et al Antibiotic sales in rural and urban pharmacies in northern Vietnam: an observational study. BMC Pharmacol Toxicol. 2014;15:6 Epub 2014/02/22. doi: 10.1186/2050-6511-15-6 ; 24555709 10.1186/2050-6511-15-6 PMC3946644

[24] Roche C, Kelliher F. Giving “Best Advice”: Proposing a framework of ommunity pharmacist professional judgement formation Pharmacy. 2014;2:74–85. doi: 10.3390/pharmacy2010074

[25] Chapman C, Braun L. The professional pharmacist and the pharmacy business. Aust Prescr. 2011;34(2):34–5.

[26] Kafle KK, Karkee SB, Shrestha N, Prasad RR, Bhuju GB, Das PL, et al Improving private drug sellers’ practices for managing common health problems in Nepal. J Nepal Health Res Counc. 2013;11(24):198–204. 24362611

[27] Ahmad A, Patel I, Mohanta G, Balkrishnan R. Evaluation of self medication practices in rural area of town sahaswan at northern India. Ann Med Health Sci Res. 2014;4(Suppl 2):S73–8. Epub 2014/09/04. doi: 10.4103/2141-9248.138012 25184092 10.4103/2141-9248.138012 PMC4145522

[28] Ansari M. Evaluation of the most commonly dispensed antibiotics among the pharmacies located in and around National Medical College Teaching Hospital, Birgunj, Nepal. Indian Journal of Pharmacy Practice. 2013;6(3):62–4.

