# Supplementary material for: Evaluation of community pharmacies regarding dispensing practices of antibiotics in two districts of central Nepal
Source: PLoS One. 2017 Sep 26;12(9):e0183907. doi: 10.1371/journal.pone.0183907 (PMC5614431; doi:10.1371/journal.pone.0183907)
Supplement: S1 File — (DOC) [file pone.0183907.s001.doc]

**Survey Questionnaire**

Q1. Age:……..Yrs

Q2. Gender: 1🞏 Male 2🞏 Female

Q3. Work experience: 1🞏 < 5 yrs 2🞏 5-10 yrs 3🞏 >10 yrs

Q4. Location of pharmacy: 1🞏 Inside city 2🞏 Near hospital 3🞏 Periphery

Q5. District in which pharmacy is situated: 1🞏 Bara 2🞏 Parsa

Q6. Qualification: 1🞏 Primary 2🞏 Secondary 3🞏 Higher secondary

4🞏 Diploma in pharmacy 5🞏 Bachelor in pharmacy

6🞏 Community Medical Assistant (CMA)

Q7. Registration status of the pharmacy: 1🞏 Based on orientation training

2🞏 Pharmacist borrowed license 3🞏 Not registered 4🞏 Pharmacist's license

Q8. Number of antibiotics encountered per prescription:

1🞏 One 2🞏 Two 3🞏 Three

Q9. Do you dispense antibiotics without prescription? 1🞏 Yes 2🞏 No

Q10.What will you do if patient ask for antibiotics without prescription?

1🞏 Ask for prescription 2🞏 Dispense without prescription

3🞏 Others (please specify)…………………………

Q11. Do you take feedback after dispensing antibiotics? 1🞏 Yes 2🞏 No

Q12. What will you do if the prescribed brand of antibiotic is not available in your pharmacy?

1🞏 Replace with other brand 2🞏 Refer to other pharmacy

3🞏 Refer to the prescriber

Q13. What advice do you give to patient about using antibiotics?

1🞏 Complete full course 2🞏 Adhere to dosage regimen 3🞏 None

Q14. What will you do if the patient has insufficient money?

1🞏 Replace with cheaper brand 2🞏 Reduce the quantity of antibiotics

3🞏 Ask to arrange for more money

Q15. From which source do you get information about antibiotics?

1🞏 Medicine Index (MI) 2🞏 Medical Representatives (MR)

3🞏 MI + MR 4🞏 None 5🞏 Others (please specify)…………

Q16. Which antibiotics do you dispense more frequently? *(You can tick more than one response)*

1🞏 Amoxicillin 2🞏 Ampicillin+Cloxacillin 3🞏 Amoxicillin +Clavulanic acid 4🞏 Cefixime 5🞏 Ceftriaxone 6🞏 Ciprofloxacin

7🞏 Ofloxacin 8🞏 Azithromycin 9🞏 Roxithromycin

10🞏 Cotrimoxazole 11🞏 Others (please specify)…………………………..

Q17. On average, how many antibiotics do you dispense every day?

1🞏 1 to 10 2🞏 11 to 20 3🞏 21 to 30 4🞏 31 to 40

5🞏 41 to 50 6🞏 51 to 60 7🞏 Above 60
